# Untargeted metabolomics reveals that declined PE and PC in obesity may be associated with prostate hyperplasia

**DOI:** 10.1371/journal.pone.0301011

**Published:** 2024-04-19

**Authors:** Guorui Fan, Xiaohai Guan, Bo Guan, Hongfei Zhu, Yongchao Pei, Chonghao Jiang, Yonggui Xiao, Zhiguo Li, Fenghong Cao

**Affiliations:** 1 Clinical Medical College, North China University of Science and Technology, Tangshan, China; 2 School of Public Health, North China University of Science and Technology, Tangshan, China; Jan Biziel University Hospital No 2 in Bydgoszcz: Szpital Uniwersytecki Nr 2 im dr Jana Biziela w Bydgoszczy, POLAND

## Abstract

**Background:**

Recent studies have shown that obesity may contribute to the pathogenesis of benign prostatic hyperplasia (BPH). However, the mechanism of this pathogenesis is not fully understood.

**Methods:**

A prospective case–control study was conducted with 30 obese and 30 nonobese patients with BPH. Prostate tissues were collected and analyzed using ultra performance liquid chromatography ion mobility coupled with quadrupole time-of-flight mass spectrometry (UPLC-IMS-Q-TOF).

**Results:**

A total of 17 differential metabolites (3 upregulated and 14 downregulated) were identified between the obese and nonobese patients with BPH. Topological pathway analysis indicated that glycerophospholipid (GP) metabolism was the most important metabolic pathway involved in BPH pathogenesis. Seven metabolites were enriched in the GP metabolic pathway. lysoPC (P16:0/0:0), PE (20:0/20:0), PE (24:1(15Z)/18:0), PC (24:1(15Z)/14:0), PC (15:0/24:0), PE (24:0/18:0), and PC (16:0/18:3(9Z,12Z,15Z)) were all significantly downregulated in the obesity group, and the area under the curve (AUC) of LysoPC (P-16:0/0/0:0) was 0.9922. The inclusion of the seven differential metabolites in a joint prediction model had an AUC of 0.9956. Thus, both LysoPC (P-16:0/0/0:0) alone and the joint prediction model demonstrated good predictive ability for obesity-induced BPH mechanisms.

**Conclusions:**

In conclusion, obese patients with BPH had a unique metabolic profile, and alterations in PE and PC in these patients be associated with the development and progression of BPH.

## 1. Introduction

Benign prostatic hyperplasia (BPH) is one of the most common diseases in older men, accounting for approximately 42% of all BPH cases by age 50 years, 80% by age 80 years, and 90% by age 85 years [1, 2]. Patients with BPH typically present with several lower urinary tract symptoms (LUTS), including urinary frequency and urgency, nocturia, urinary hesitancy, and decreased urination, which can lead to urinary dysfunction and have a significant negative impact on quality of life [3]. BPH, considered a complex inflammatory state, is significantly influenced by body mass index (BMI) and waist circumference (WC). In addition, it has been shown that obesity leads to metabolic disorders that increase the level of circulating proinflammatory factors and systemic oxidative stress, thereby promoting immune cell infiltration and leading to increased severe prostate enlargement and LUTS [4–6]. Moreover, studies have shown that increased obesity promotes the aromatization of circulating testosterone to estrogen and that an altered balance of testosterone and estrogen in the prostate tissue may contribute to the pathogenesis of BPH [7, 8]. Several studies have shown that obesity not only increases the risk of prostate cancer but also significantly increases the malignancy of prostate cancer. Systemic inflammation, alterations in the insulin and IGF-1 axis, and variations in sex hormone levels and adipokines are among the underlying mechanisms of the relationship between obesity and prostate cancer [9]. These mechanisms may be involved in epithelial to mesenchymal transformation into a malignant phenotype that promotes invasiveness, aggressiveness, and metastatic potential of prostate cancer [10]. Although progress has been made on elucidating the causes associated with obesity-induced prostate damage, the mechanisms underlying the relationship between BPH and obesity remain unclear. Therefore, there is an urgent need to explore the mechanisms associated with obesity-induced BPH to develop new strategies for BPH prevention and treatment.

Metabolomics, a new branch of systems biology after genomics, transcriptomics, and proteomics [11, 12], can be used to detect many endogenous metabolites, including organic acids, amino acids, fatty acids, sugars, and cholesterol, by analyzing body fluids and tissues. Thus, it can be used to detect altered pathological pathways in diseases [13]. Metabolomics, combined with certain advanced analytical techniques (such as nuclear magnetic resonance and mass spectrometry (MS)) and high-throughput bioinformatics tools, has been widely used to study urological diseases [14, 15]. However, the nuclear magnetic resonance and general MS analytical techniques have several disadvantages, including low relative sensitivity and narrow dynamic range of detection [16, 17]. Ultra performance liquid chromatography ion mobility spectroscopy quadrupole-time-of-flight mass spectrometry (UPLC-IMS-Q-TOF) combines UPLC separation with the ion structure information provided by collision cross section to derive more accurate structural inferences with greater sensitivity and speed [18, 19]. However, no study has evaluated the metabolomic profile of obesity-induced BPH.

In the current study, a prospective case–control study was conducted with 30 obese and 30 nonobese patients with BPH. The prostate tissues of the patients were collected to analyze the metabolomics of the prostate using UPLC-IMS-Q-TOF. The study results may provide new clues to identify the potential mechanisms of BPH pathogenesis and develop the prevention and treatment strategy of obesity-induced BPH.

## 2. Materials and methods

### 2.1. Patient recruitment and sample collection

In this study, 60 patients with BPH were recruited from June 2022 to January 2023, including 30 obese patients with BMI ≥ 26 kg/m^2^ and WC ≥ 90 cm and 30 nonobese patients with BMI ≤ 20 kg/m^2^ and WC ≤ 80 cm. The patients were recruited from the Affiliated Hospital of North China University of Technology. Patient characteristics are shown in Table 1. These patients were included in the study after BPH diagnosis using trans-ultrasound prostate puncture pathology. To avoid the influence of the surgical approach on the results, prostate tissue was obtained via transurethral resection of the prostate. All patients were free of prostatitis, prostate abscess, prostate cancer, and tumors of other tissues. To avoid the effects of associated metabolic diseases, patients with other metabolic diseases, such as diabetes mellitus and hyperlipidemia, were not included. The included patients signed an informed consent form prior to participation in the study, which was conducted in accordance with the Declaration of Helsinki and approved by the ethics committee of the North China University of Technology. Prostate tissues were obtained within 30 min of surgery and immediately stored at −80°C.

**Table 1:** Demographic characteristics of the studied groups.

Characteristics	obese	Non-obese
	group	group
	(n = 30)	(n = 30)
Age (years)	67±7	65±5
BM (kg/m^2^)	29.4±3.4	19.4±2.6 **
WC (cm)	98±8	76±4 **
TG (mmol/L)	1.40±0.13	1.36±0.16
TC (mmol/L)	4.81±0.34	4.64±0.30
PSA(ng/ml)	5.325±5.035	3.675±2.885
Prostate Volume (ml)	93.15±12.35	49.15±11.65 **
IPSS	28.5±3.5	21±5 **

Table note:

** on behalf of *p* < 0.01, prostate hyperplasia clinical features statistically difference between the two groups. Abbreviations: TG, total cholesterol; TC, triglycerid; BMI, body mass index; WC, waist circumference; PSA, prostate specific antigen; IPSS, international prostate symptom score.

### 2.2. Sample preparation

Aqueous and organic metabolic extracts were obtained using a modified and optimized protocol, which was originally outlined by Want et al. [20]. The prostate tissue samples were homogenized with a precooled mixture of methanol and water (1:1, v/v) at a ratio of 0.03 mL/mg for 2 min and then sonicated for 30 s. After centrifugation (12,000 × *g*, 10 min), the supernatant and precipitate were collected into 2-mL centrifuge tubes and used to extract the aqueous and organic components, respectively. The supernatant was concentrated under vacuum using Tokyo EYELA CVE-3100 centrifugal concentrator, and the dried powder (180 min, 45°C) was used as the aqueous extract. The precipitate was mixed with precooled dichloromethane/methanol (3:1, v/v) at a ratio of 0.03 mL/mg for 2 min, and after centrifugation (12,000 × *g*, 10 min), the supernatant containing the organic component was dried under vacuum to make a powder of the organic component. Both extracts were stored at −80°C for LC–MS analysis. Prior to LC–MS analysis, both extracts were redissolved in 7.2 μL/mg methanol/water (1:1, v/v), followed by centrifugation twice (12,000 × *g*, 4°C, 10 min) to remove the precipitate and obtain the final samples. Quality control (QC) was performed by mixing different samples. QC injections were performed periodically (once every 10 samples) to monitor the experimental stability.

### 2.3. UPLC-IMS-Q-TOF analysis

For excellent detection sensitivity and high metabolite screening, the protocol reported by Want et al. was followed for analysis. The aqueous and organic metabolic extracts were analyzed using Waters I-Class Identification UPLC (Waters, Elstree, UK) and Vion-IMS-QToF (Waters, Elstree, UK). For analysis, two different columns were used for the aqueous and organic metabolic extracts, respectively. The HSST3 column (2.1 × 100 mm, 1.8 μm) (Waters, Ireland) was used to analyze the aqueous metabolic extract: mobile phase A consisted of 0.1% formic acid and mobile phase B comprised a methanol solution containing 0.1% formic acid. The gradient was set to 10%–0.1% A and 90%–99.9% B for 12–21 min and 0.1%–99.9% A and 99.9%–0.1% B for 23–24 min. The BEH C8 column (2.1 × 100 mm, 1.7 μm) (Waters, Ireland) was used to analyze the organic metabolic extract: mobile phase A consisted of 0.1% formic acid and mobile phase B comprised methanol and isopropanol in a ratio of 85:15 (v/v) with 0.1% formic acid. The gradient was set to 10%–0.1% A and 90%–99.9% B for 17–29 min and 0.1%–25% A and 99.9%–75% B for 32–33 min. The flow rates of the two different columns were set at 0.4 mL/min at 50°C, with the injection volume of 5 μL and sample temperature of 4°C.

ESI detection was performed in the positive and negative detection modes, and the main parameters of the ESI source were as follows: capillary voltages of 2.4 kV (ESI−) and 3.2 kV (ESI+), m/z range of 50–1000, source temperature of 120°C, and desolventization gas temperature of 350°C. The flow rates of the cone gas and desolventization gas were set to 25 and 900 L/h, respectively.

### 2.4. Data processing and analysis

Raw data were acquired using the Waters UNIFI 1.8.1 software and processed using the Progenesis QI 3.0 software (Waters, Nonlinear Dynamics, Newcastle, UK) to perform peak integration, calibration, and quantitation for each metabolite. Normalized data matrix was exported from QI to identify metabolites with variable importance in projection > 1, P-value < 0.05, and fold change > 2 as differential metabolites. The identified differential metabolites were annotated using the Kyoto Encyclopedia of Genes and Genomes (KEGG) database (http://www.genome.jp/kegg/) and the Human Metabolome Database (HMDB) (http://www.hmdb.ca/) and analyzed using SIMCA 14.1 (Umetrics AB, Sweden) for principal component analysis (PCA) and orthogonal partial least squares discriminant analysis (OPLS-DA). The metabolic pathway enrichment and heat mapping of the differential metabolites were performed using Metabolic Analyst (https://www.metaboanalyst.ca/). Pearson’s correlation and hierarchical (average linkage) clustering were performed for untargeted metabolomic analyses. Receiver operating characteristic (ROC) curves and graphs were generated using GraphPad Prism (version 8.0.1) and OriginPro 2021.

## 3. Results

### 3.1. Patient characteristics

**Fig 1:**
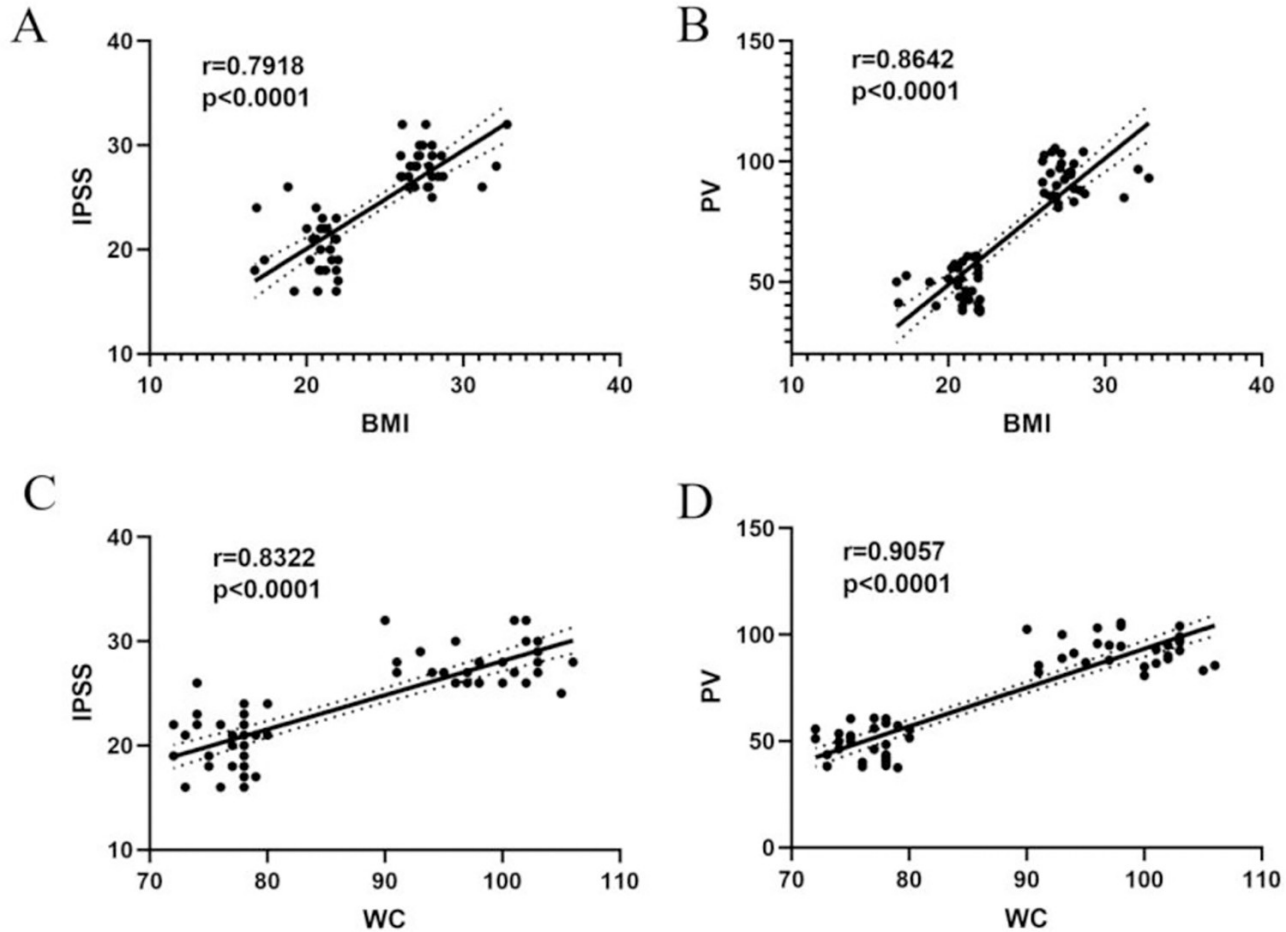
The correlation between the degree of obesity and clinical features. (**A)** The correlation between BMI and IPSS; (B) The correlation between BMI and PV; (C) The correlation between WC and IPSS; (D) The correlation between WC and PV. Abbreviations: BMI, body mass index; WC, waist circumference; PV, prostate volume; IPSS, international prostate symptom score.

Table 1 shows the demographic characteristics of the two patient groups, including age, BMI, WC, total cholesterol (TC), triglyceride (TG), prostate-specific antigen (PSA), prostate volume (PV), and international prostate symptom score (IPSS). No significant difference was observed between the obese and nonobese groups in terms of age (67 vs. 65 years, *p* > 0.05), TG (1.40 vs. 1.36, *p* > 0.05), TC (4.81 vs. 4.64, *p* > 0.05), and PSA (5.325 vs. 3.675, *p* > 0.05), almost excluding the effects of age and lipids on the experimental results. However, there were significant differences in BMI (29.4 vs. 19.4, *p* < 0.01), WC (98 vs. 76, *p* < 0.01), PV (93.15 vs. 49.15, *p* < 0.01), and IPSS (28.5 vs. 21, *p* < 0.01). To investigate the relationship of patient BMI and WC with PV and IPSS, correlation analysis was performed. The results showed that BMI was significantly positively correlated with IPSS (r = 0.7918; *p* < 0.00001), BMI was significantly positively correlated with PV (r = 0.8642; *p* < 0.0001), WC was significantly positively correlated with IPSS (r = 0.8322; *p* < 0.0001), and WC was significantly positively correlated with PV (r = 0.9057 *p* < 0.0001) (Fig 1).

### 3.2. Multivariate analysis of prostate metabolites in obese and nonobese patients

**Fig 2:**
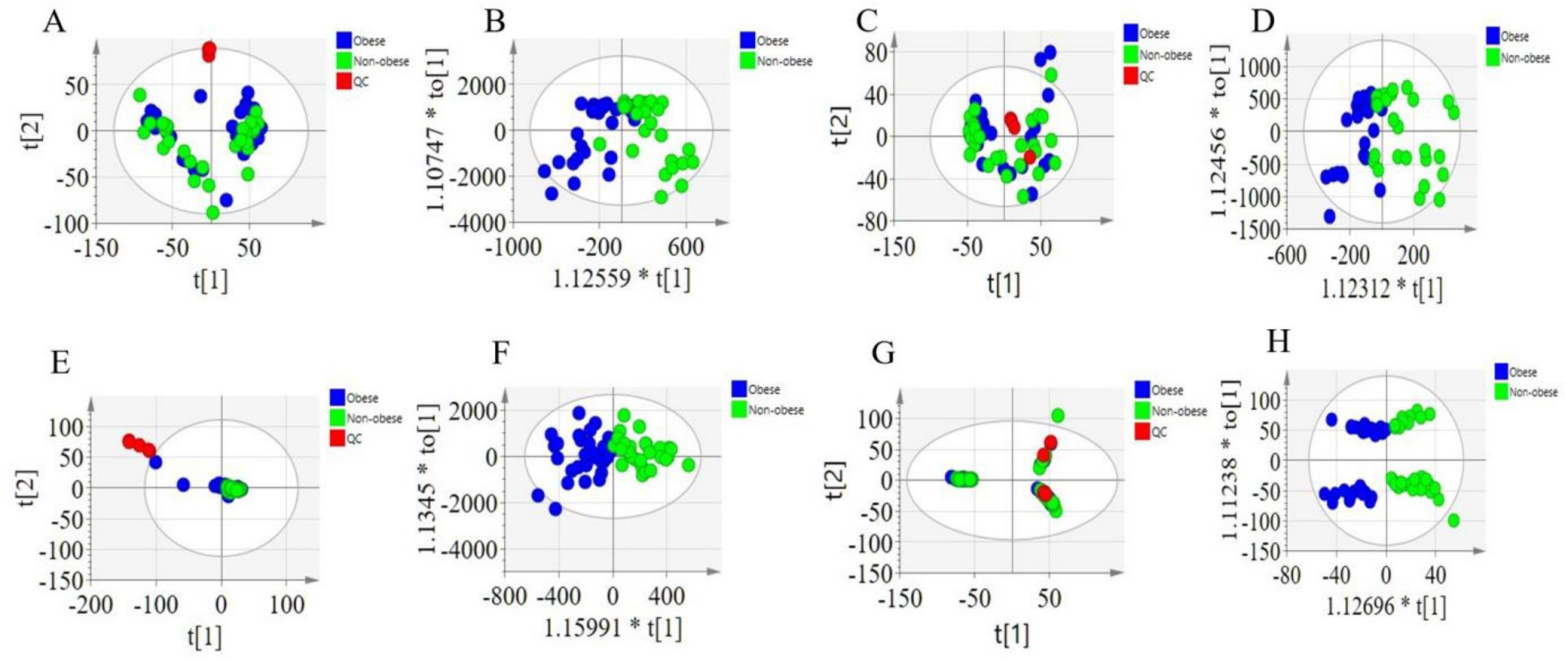
PCA and OPLS-DA of aqueous extracts and organic matter. Multivariate statistical analysis of organic phase extracts, ESI (+), **(A)** PCA score plot, **(B)** OPLS-DA score plot;ESI (-), **(C)** PCA score plot, **(D)** OPLS-DA score plot. Multivariate statistical analysis of aqueous extracts, ESI (+), **(E)** PCA score plot, **(F)** OPLS-DA score plot;ESI (-), **(G)** PCA score plot, **(H)** OPLS-DA score plot.

The nontargeted metabolomic analysis of the prostate tissue was performed using HSS T3 and BEH C8 columns, detecting 26,882 and 40,465 features, respectively. Unsupervised PCA was used to identify outlier samples from the sample features to assess data quality and determine differences between the metabolic profiles of patients in the obese and nonobese groups. The results of the PCA score plots in the positive and negative ionization modes showed that both study groups achieved relatively good separation. Moreover, the QC samples formed a tight cluster with good reproducibility and instrumental stability, validating the quality of the obtained results (Fig 2A, 2C, 2E and 2H). In addition, a supervised OPLS-DA classification model with orthogonal components was developed with the characteristics of all samples individually to exclude less relevant variables. The OPLS-DA scoring plots showed a clear distinction among categories for positive and negative ion patterns (Fig 2B, 2D, 2F and 2G). Thus, a good distinction among the metabolic characteristics of all patients in the obese and nonobese groups was obtained, which suggested that the metabolites can differentiate the obese group from the nonobese group.

### 3.3. Differential metabolite screening and clinical correlation analysis

**Fig 3:**
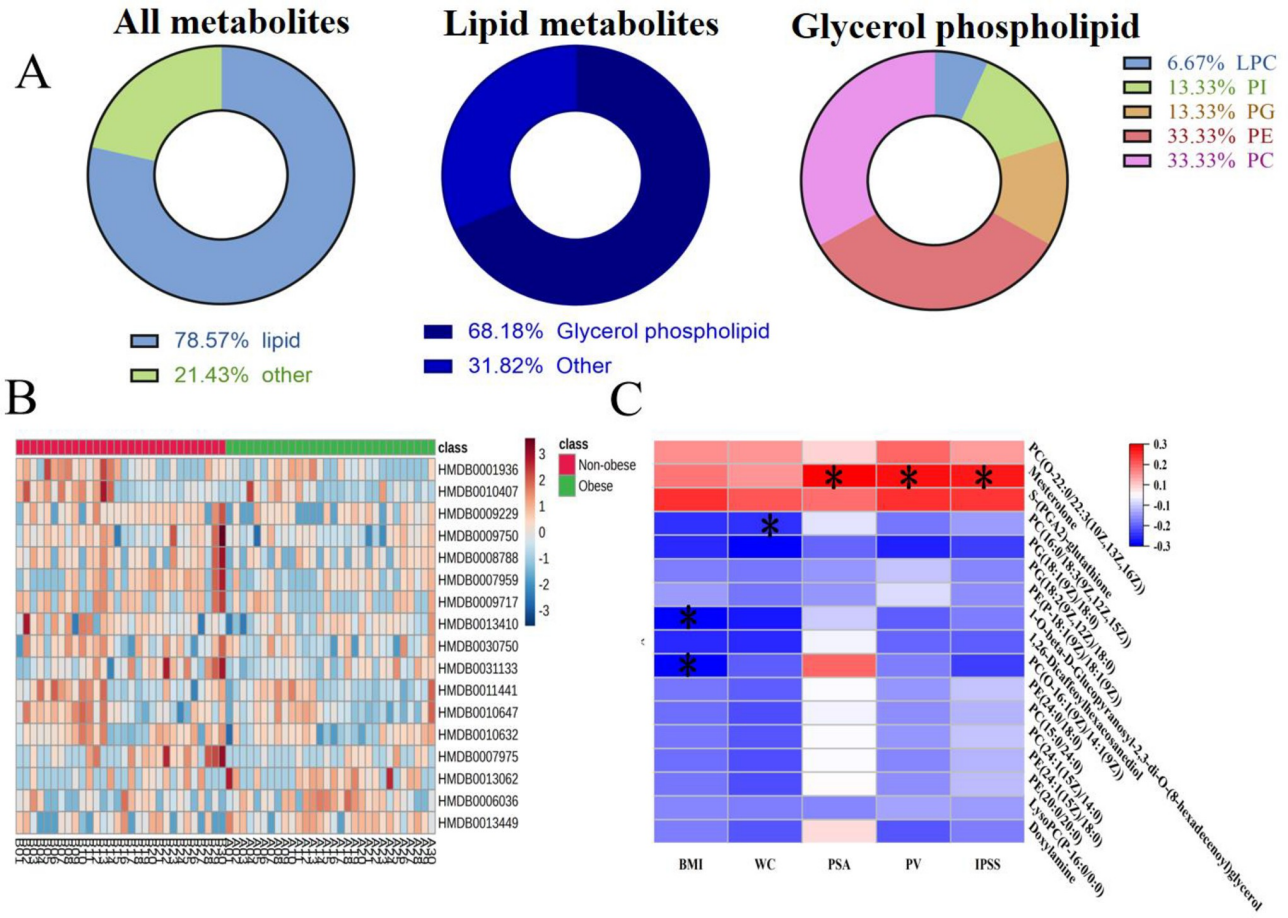
The analysis of the differences of metabolites. **(A)** Obese and non-obese patients with hyperplasia of prostate metabolic differences in content of rough classification. **(B)** Heatmap of differential metabolites clustering between in the BPH obese and Non-obese groups. **(C)** Spearman correlation matrix of the selected lipids with clinical variables. Adjusted p-values with a cutoff at <0.05 are marked with *. Positive correlations are in red, and negative correlations are in blue. Abbreviations: LPC, lysophosphatidyl choline; PI, glycerophosphateinositol; PG, glycerophosphateglycerol; PE, glycerophosphatidylethanolamine; PC, glycerophosphateidylcholine; BMI, body mass index; WC, waist circumference; PSA, prostate specific antigen; PV, prostate volume; IPSS, international prostate symptom score.

A total of 17 differential metabolites were identified with high confidence (Table 2). Differential expression of metabolic differentials is detailed in S1 Table. Of the 17, 78.57% were lipid metabolites, and approximately 68.18% of the lipid metabolites were glycerophospholipid (GP) metabolites. The GP metabolites were further classified into phosphatidylethanolamine (PE; 33.33%), phosphatidylcholine (PC; 33.33%), phosphatidylglycerol (PG; 13.33%), phosphatidylinositol (PI; 13.33%), and lysophosphatidylcholine (LPC; 6.67%) (Fig 3A). The heatmap showed a clear distinction among the 17 differential metabolites, and compared with the obese group, the level of 3 differential metabolites was downregulated and that of 14 metabolites was upregulated (Fig 3B).

**Table 2:** Differential metabolites for distinction of BPH obese and Non-obese.

HMBD ID	name	Measured m/z	RT (MIN)	Adducts	Measured CCS(Å2)	Predicted CCS(Å2)	Anova (p)	Fold Change	VIP	Trend
HMDB0001936	Doxylamine	271.1796719	0.54	M+H	162.3	162.2	0.032	11.47	1.42	↑
HMDB0010407	LysoPC(P-16:0/0:0)	480.3424699	2.02	M+H	232.3	229.6	0.047	2.81	2.64	↑
HMDB0009229	PE(20:0/20:0)	804.6454902	11.52	M+H	321.8	301.7	0.043	23.19	1.20	↑
HMDB0009750	PE(24:1(15Z)/18:0)	830.6625108	11.78	M+H	331.3	300.4	0.025	13.89	2.26	↑
HMDB0008788	PC(24:1(15Z)/14:0)	816.6482772	11.81	M+H	324.0	293.7	0.024	138.18	1.50	↑
HMDB0007959	PC(15:0/24:0)	854.6630646	12.27	M+Na	335.8	303.5	0.021	27.86	1.32	↑
HMDB0009717	PE(24:0/18:0)	832.6786126	13.43	M+H	336.3	303.5	0.019	243.70	1.52	↑
HMDB0013410	PC(O-16:1(9Z)/14:1(9Z))	686.5133655	8.53	M-H	270.2	261.1	0.039	5.94	1.15	↑
HMDB0030750	1,26-Dicaffeoylhexacosanediol	721.4837602	8.94	M-H	269.6	266.7	0.024	3.98	1.35	↑
HMDB0031133	1-O-beta-D-Glucopyranosyl-2,3-di-O-(8-hexadecenoyl)glycerol	725.5124148	10.32	M-H	267.5	270.4	0.049	14.62	8.23	↑
HMDB0011441	PE(P-18:1(9Z)/18:1(9Z))	726.5433841	10.38	M-H	280.1	267.6	0.042	3.74	3.12	↑
HMDB0010647	PG(18:2(9Z,12Z)/18:0)	773.5340644	10.55	M-H	283.6	288.6	0.042	3.74	7.53	↑
HMDB0010632	PG(18:1(9Z)/18:0)	775.5486511	11.77	M-H	287.8	283.7	0.041	3.34	5.43	↑
HMDB0007975	PC(16:0/18:3(9Z,12Z,15Z))	753.5435431	13.07	M-H	279.7	277.7	0.007	156.49	1.68	↑
HMDB0013062	S-(PGA2)-glutathione	642.3077953	8.53	M+H	233.9	253.0	0.039	20.44	1.34	↓
HMDB0006036	Mesterolone	303.2296032	8.93	M+Na	181.5	180.9	0.021	2.45	1.93	↓
HMDB0013449	PC(O-22:0/22:3(10Z,13Z,16Z))	881.7575977	10.32	M+H	347.3	318.0	0.022	2.04	1.77	↓

Table note: Trend represents the trend of differential metabolites in non-obese individuals.

Correlation analysis revealed that merodesmosine was significantly positively correlated with PSA, PV, and IPSS (*p* < 0.05). Moreover, PG (18:1(9Z)/18:0) was significantly negatively correlated with WC (*p* < 0.05), whereas 1-O-beta-D-Glucopyranosyl-2,3-di-O-(8-hexadecenoyl)glycerol and PC (O-16. 1(9Z)/14:1(9Z)) were significantly negatively correlated with BMI (*p* < 0.05) (Fig 3C).

### 3.4. Metabolomic analysis

**Fig 4:**
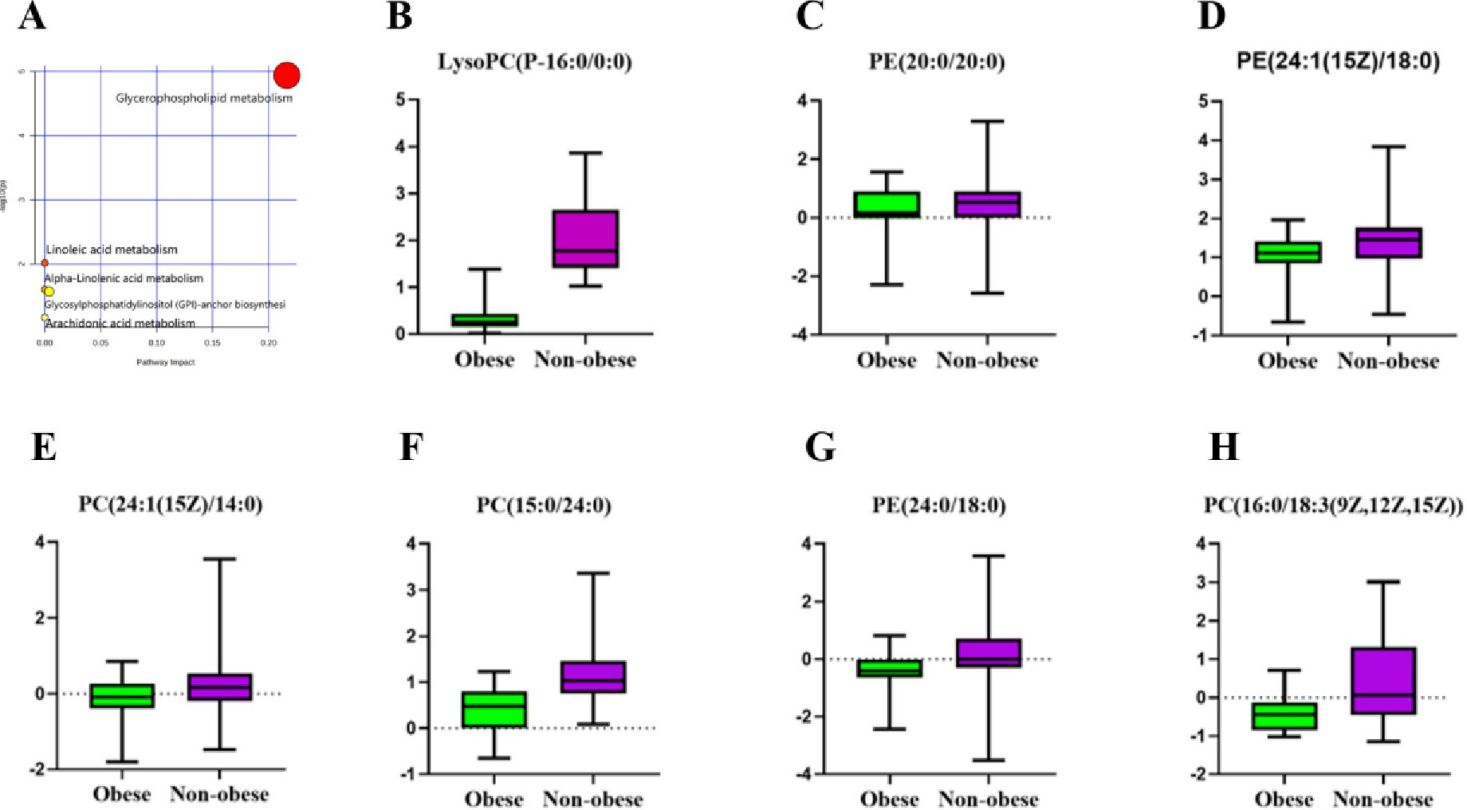
Differential metabolite enrichment pathways and differential expression of glycerophospholipid metabolism-enriched metabolites. **(A)** Bubble plots of differential metabolite pathways in the BPH obese and Non-obese groups. **(B-H)** LysoPC(P-16:0/0:0),PE(20:0/20:0),PE(24:1(15Z)/18:0),PC(24:1(15Z)/14:0),PC(15:0/24:0), PE(24:0/18:0) and PC(16:0/18:3(9Z,12Z,15Z)) of expression differences of BPH obese and Non-obese groups.

MetaboAnalyst 5.0 was used for the enrichment and topological analyses of the pathways with the 17 identified differential metabolites. According to the KEGG database, five metabolic pathways were identified, suggesting that these pathways are involved in the metabolic network of obesity-induced BPH (Fig 4H). The GP metabolism pathway with an impact of >0.1 was considered the most significant metabolism pathway (Table 3).

**Table 3:** Different metabolic enrichment pathways between obese and non-obese groups.

Pathway ID	Pathway Name	Match Status	RAW p	FDR	Impact
hsa00564	Glycerophospholipid metabolism	3/36	1.1526E-5	9.6822E-4	0.21631
hsa00591	Linoleic acid metabolism	1/5	0.0096524	0.4054	0.0
hsa00592	alpha-Linolenic acid metabolism	1/13	0.024967	0.56427	0.0
hsa00563	Glycosylphosphatidylinositol (GPI)-anchor biosynthesis	1/14	0.02687	0.56427	0.00399
hsa00590	Arachidonic acid metabolism	1/36	0.068115	1.0	0.0

Table note: Pathway ID: KEGG pathways; Match status: Metabolites involved in the pathway. Data before "/" indicates the number of metabolites currently involved in the pathway; The number after "/" indicates the total number of metabolites in the current pathway; Impact value: represents the overall importance score of the pathway, with a total score of 1, which can be calculated based on the relative position of metabolites in the pathway.

**Fig 5:**
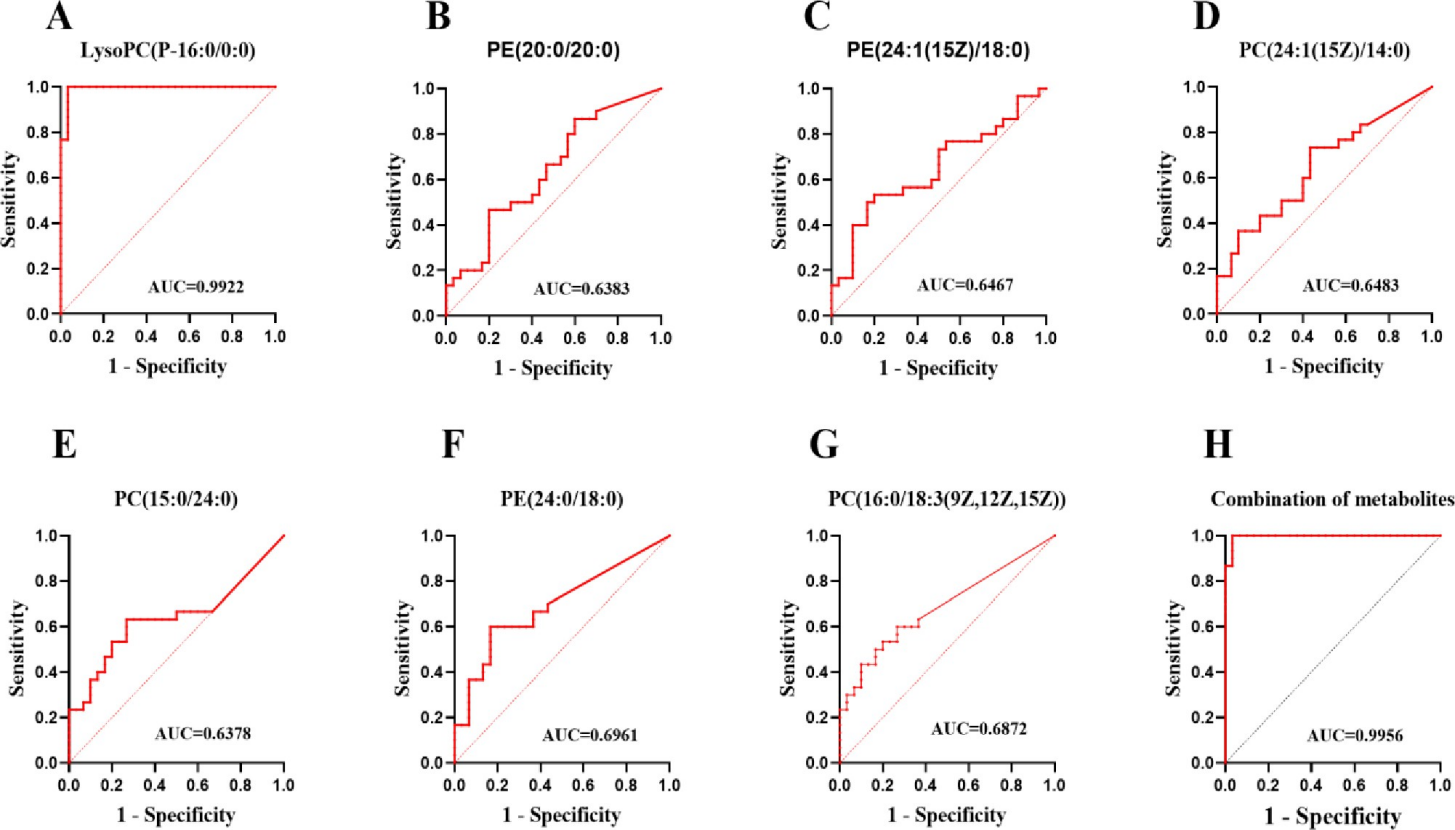
Obese and non-obese group metabolic differences and combination of the ability to predict. (A-G) ROC plot with for distinction of BPH obese and Non-obese based on LysoPC(P-16:0/0:0),PE(20:0/20:0), PE(24:1(15Z)/18:0), PC(24:1(15Z)/14:0), PC(15:0/24:0), PE(24:0/18:0) and PC(16:0/18:3(9Z,12Z,15Z)). (H) ROC plot with for distinction of BPH obese and Non-obese based on combined metabolites panel.

The GP metabolism was mainly enriched in seven differential metabolites, namely, LysoPC (P-16:0/0:0), PE (20:0/20:0), PE (24:1(15Z)/18:0), PC (24:1(15Z)/14:0), PC (15:0/24:0), PE (24:0/18:0), and PC (16:0/18:3(9Z,12Z,15Z)), which can be classified into PC and PE. All seven enriched differential metabolites were downregulated in the obese group (Fig 4A–4G). A multivariate ROC curve analysis was performed to assess the predictive ability of the seven differential metabolites in obesity-induced BPH. The area under the curve (AUC) was used to test the reliability of the differential metabolites, and the results showed that LysoPC (P-16:0/0:0) had an AUC value of 0.9922, demonstrating a high predictive value for identifying obesity-induced BPH (Fig 5A–5G). Studies have shown that a combination of differential metabolites is more valuable for predicting disease progression than using a single differential metabolite. Thus, a panel comprising LysoPC (P-16:0/0:0), PE (20:0/20:0), PE (24:1(15Z)/18:0), PC (24:1(15Z)/14:0), PC (15:0/24:0), PE (24:0/18:0), and PC (16:0/18:3(9Z,12Z,15Z)) exhibited the best predictive ability with an ROC area of 0.9956 for the testing dataset (Fig 5H).

## 4. Discussion

In this prospective case–control study, the correlation of BMI or WC with PV or IPSS was assessed, and the results revealed that BMI and WC had a significant positive correlation with both PV and IPSS. Most studies have shown that obesity increases BPH risk, with those with an obese WC (>109 cm) being 38% more likely to undergo BPH surgery than those without (WC < 89 cm) [21]. The results of a case–control study on 3000 Italian men revealed an association between obesity and BPH, with men with a minimum BMI of 23.7 kg/m^2^ being 56% more likely to develop BPH than men with a maximum BMI of 20.7 kg/m^2^ [22]. These studies are consistent with the present study results that obesity was significantly associated with the development of and progression of BPH.

In the current study, 17 differential metabolites were identified in the obese and nonobese BPH groups, of which mesterolone, an androgenic steroid, was significantly positively correlated with PSA, PV, and IPSS. In one study, testosterone undecanoate was administered to 23 gonadally normal males for 8 months, which led to a 12% increase in the mean prostate volume [23]. This hypothesized link between testosterone and prostate growth has led to the general belief that testosterone exacerbates BPH/LUTS, in line with the current study findings. Thus, the increase in mesterolone level because of obesity may induce the progression of obesity-induced BPH. However, few studies have evaluated the relationship between obesity and BPH, which warrants further research.

The pathway analysis results suggest that the most important in the obesity-induced BPH mechanism is GP metabolism. Of note, seven differential metabolites involved in GP metabolism were downregulated in the obese group. The results suggest that the seven metabolites and combinations of differential metabolites had a good predictive ability for obesity-induced BPH. The best predictive ability was shown by LysoPC (P16: 0/0:000), a precursor of lysophosphatidic acid (LPA), which is a GP involved in various biological processes, such as fibrosis, inflammation, atherosclerosis, and obesity [24–28]. Studies have shown that LPA induces pulmonary, renal, and hepatic fibroses through epithelial cell death, vascular leakage, migration, and fibroblast proliferation [25, 29–31]. However, in BPH, prostatic interstitial fibrosis is considered an important cause of accelerated BPH progression [32, 33]. The present results revealed LysoPC downregulation in tissues from obese patients with BPH, which suggests that the LysoPC to LPA conversion pathway plays a key role in BPH. Of note, the differential metabolites enriched in this study mainly consisted of PE and PC. Research has shown that obese patients have lower phospholipid levels than normal-weight patients [34], which is consistent with PE and PC downregulation observed in this study. Therefore, PE and PC reduction may be an important reason for obesity-induced BPH [35]. Unfortunately, there is limited research on the relationship between PE and PC and human disease. However, there is growing evidence that phospholipids are associated with chronic metabolic disorders. Typically lower phospholipid levels are associated with disease onset and progression. For example, hypertension, coronary artery disease, and myocardial infarction are highly associated with lower serum levels of acetylated phospholipids [36–39]. Similarly, the ability of phospholipid supplementation to attenuate atherosclerosis in mouse models confirms the findings.

**Fig 6:**
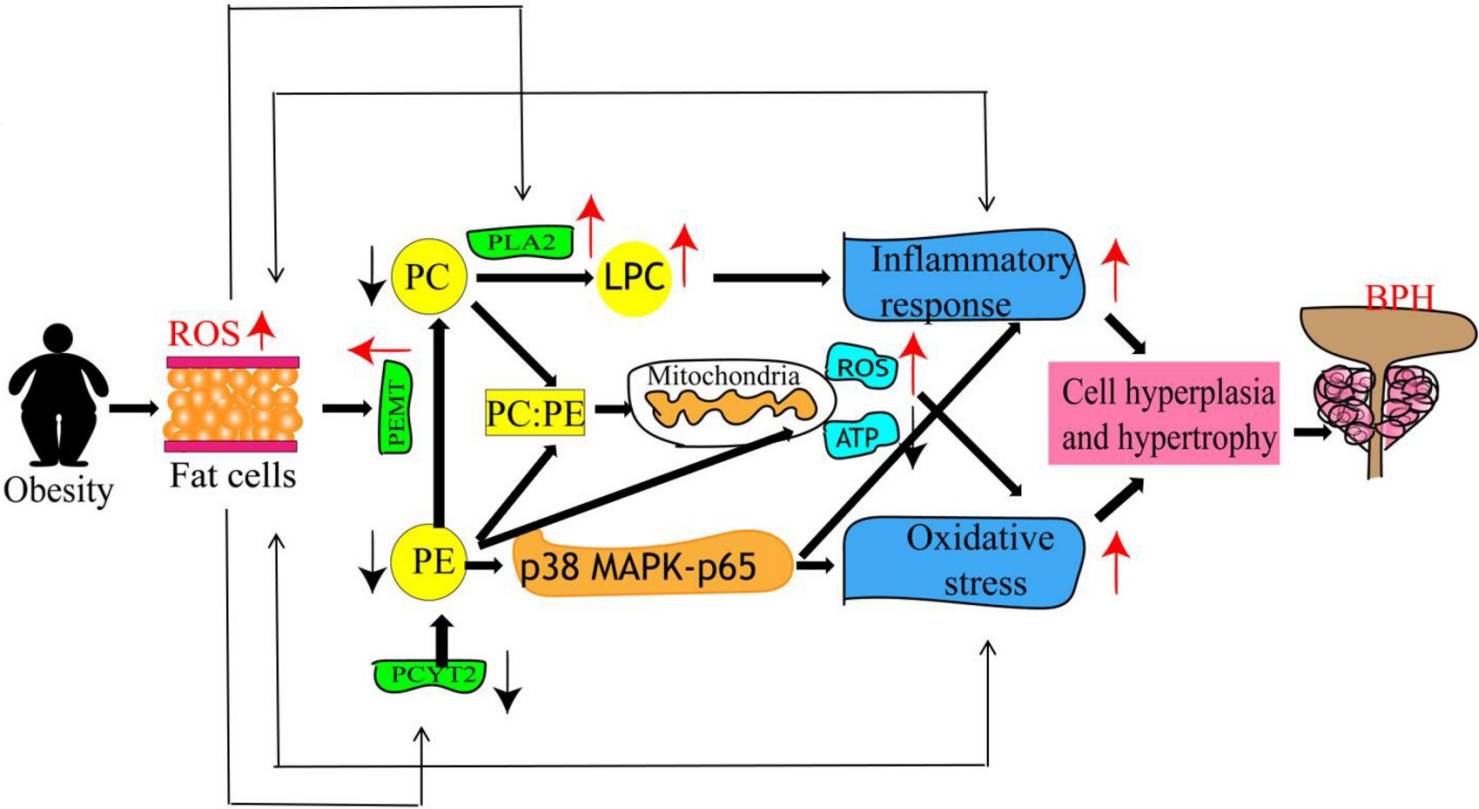
Mechanisms associated with PE and PC alterations promoting prostatic hyperplasia. Its mechanism is as follows: In obese patients, PCYT2 is reduced and PEMT is elevated, resulting in decreased PE synthesis and increased conversion and thus decreased expression.PE content reduce the levels of increased oxidative stress leading to loss of the prostate.PE can also lead to reduce damage mitochondrial reactive oxygen species (ROS), ATP content is lower. In addition, Obesity can induce elevated PLA2 to promote the conversion of PC to LPC resulting in decreased expression., PLC promotes prostate damage caused by inflammation.PE and PC level changes will also affect PC: PE, PC:PE changes will also affect the change of mitochondrial function, promoting obesity again causes PE and PC alterations promoting inflammatory prostate injury.

PE is an abundant membrane phospholipid and an important precursor of PC [40]. It performs various cellular functions and is essential for cell cycle [41], apoptosis [42], oxidative phosphorylation [43, 44], mitochondrial biogenesis, and autophagy [45, 46]. PE is synthesized primarily through the CDP-ethanolamine Kennedy pathway, and phosphate cytidylyltransferase 2 (PCYT2) is the main regulatory enzyme for the ab initio synthesis of PE. PE can be converted to PC by PE N-methyltransferase (PEMT), and research has shown that in the adipose tissue [47], PEMT expression was positively correlated and PCYT2 expression was negatively correlated with percent fat mass and BMI [48, 49]. Therefore, an obesity-induced decrease in PCYT2 expression and an increase in PEMT expression may result in decreased PE synthesis, and increased transformation may be the cause of this decreased expression. It has been demonstrated that the addition of PE reduces the expression of proinflammatory genes by inhibiting the p38 MAPK-p65 pathway [50]. The reduction of PE may thus lead to mitochondrial dysfunction, including a decrease in mitochondrial membrane ATP level and an increase in reactive oxygen species level. Therefore, the decrease in PE may increase the expression of proinflammatory genes and enhance mitochondrial dysfunction, thereby promoting inflammatory responses and oxidative stress and contributing to the development of BPH. PC is the main phospholipid of biological membranes and exerts a very important role in biological functions [51]. PC is converted to lysophosphatidylcholine (LPC) by phospholipase A2 (PLA2) [52, 53]. Most studies have revealed a negative correlation between BMI and PC [54, 55]. The present study results showed that PC was downregulated in the obese group, which is consistent with the above findings. Moreover, it has been shown that PLA2 was positively correlated with BMI [56], which may accelerate the conversion of PC to LPC, resulting in a decrease in the expression of PC. LPC induces lymphocyte and macrophage migration, increases proinflammatory cytokine production, induces oxidative stress, and promotes apoptosis, which aggravates inflammation and contributes to BPH progression [57]. As mentioned above, the reduction in PE and PC alters the PC/PE ratio, and studies have shown that in mitochondria, changes in the PC/PE molar ratio affect energy production [57]. Moreover, changes in the PC/PE ratio in various tissues have been associated with certain metabolic disorders, such as insulin resistance and obesity [58]. Obesity, in turn, alters PE and PC expression, thereby increasing oxidative stress and inflammatory response and exacerbating BPH progression. The underlying mechanisms are shown in Fig 6.

The strengths of this study include the nonreliance on BMI as the sole indicator of obesity. Indeed, WC was included in the analysis, thereby excluding errors caused by metabolic symmetry because of central, peripheral, or visceral obesity. The analysis was limited to men without prostate cancer to ensure that prostate tissues from patients with LUTS were not malignant and only patients with BPH were included. Despite significant findings, there are some limitations to this study. Although tissue metabolomic studies are widely used to assess physiological and pathological pathways, they do not represent the complete metabolomic alterations in an organism. In addition, the sample size was relatively small and data may not have been representative, as the participants were from only one center. Therefore, additional metabolomic analyses using larger sample sizes from multiple centers are needed to validate the present study findings.

## 5. Conclusions

Taken together, we used a metabolomic approach to investigate differences in prostate tissue metabolites between obese and nonobese patients with BPH. A total of 17 differential metabolites were identified in both groups. Decreased PE and PC expression in the prostate tissues of obese patients with BPH may be associated with the development of BPH. The study results may provide novel strategies for BPH prevention and treatment.

## Supporting information

S1 Table Expression of differential metabolites between obese and non-obese groups. See S1 Table of the Additional Document for details.
(XLSX)
